# A Tabular View of the Seat of Tubercle in One Hundred and Eighty Cases of Tubercle of the Lungs in Children, with Remarks on Pulmonary Phthisis in the Young Subject

**Published:** 1845-09

**Authors:** P. Hennis Green


### PRACTICAL MEDICINE, &c.

*A tabular view of the seat of Tubercle in one hundred and eighty cases of Tubercle of the Lungs in Children, with remarks on Pulmonary Phthisis in the young subject.*—By P. Hennis Green, M. D.

In this paper the author proposes to indicate a few of the peculiarities which distinguish infantile consumption from the phthisis of adults. He gives a table of 180 cases of thoracic tubercle for the purpose of furnishing data for the history of pulmonary consumption in children. He remarks, "the main character which distinguishes the phthisis of children from that of adults is this,—in children, the tubercular deposit occupies a much larger surface of the lung, is more rapidly secreted, and is complicated with tubercular disease of other organs more frequently than in the adult. Hence children often sink under phthisis before the complaint has arrived at its third stage; while, on the other hand, the modifications produced by an extensive diffusion of tubercular matter often render the diagnosis of the disease obscure and difficult. In addition to this character, we have the peculiarities occasionally induced by excessive tuberculization of the bronchial phthisis, a form of disease altogether confined to the child."

The author notices an important modification which should guide the practitioner when he seeks to determine the existence of cavern in young children, viz., that under five years of age, the cavernous excavation is generally seated in the lower or middle lobes, and is almost always confined to one side of the chest. To show that the general effusion of tubercular matter forms a striking characteristic of phthisis in children, Dr. Green compares some of M. Louis's results with those deducible from his table.

"In 358 cases of phthisis in adults, M. Louis mentions the existence of tubercular matter in the brain or its membranes only once. In the bronchial glands, tubercles were found in about one fifth of the cases; in the mesenteric glands, in one fifth; in the liver, only twice; in the kidneys, five times in 170 cases; on the other hand, ulceration of the larynx existed in one-fourth,—ulceration of the bowels in five-sixths of the cases.

"The history of phthisis in children presents us with very different results. The brain was affected in one-ninth of the cases; the bronchial glands, in 100 out 112; the mesenteric glands in one-half; the liver in one-ninth; the kidneys in one-eighteenth; but ulceration of the larynx occurred only once; ulceration of the bowels sixteen times in the 112 cases." 356.

The physical signs are rarely as well marked as in the adult, and the young child frequently dies before the practitioner is able to decide whether the lung be actually the seat of cavern or not. The cause of this is that, in children, "the tubercular matter is widely diffused, and has implicated many important viscera; in the brain it may excite hydrocephalus or meningitis; under the serous membrane of the chest, pleurisy; in the abdomen, peritonitis; in the intestines, tubercular ulceration. These complications rarely undermine the resisting power of the little patient; diarrhœa sets in, and death ensues long before the period at which a fatal termination takes place in the adult."

Infants and children under five years of age hardly ever expectorate. They swallow everything that comes up into the mouth from the lungs. Hæmoptysis is an exceedingly rare symptom. The symptoms which constitute hectic fever in adults are seldom present together in any marked degree.

The bronchial glands were more or less affected in one hundred out of one hundred and twelve cases. In a few of these cases only were the glands sufficiently enlarged to produce symptoms through their mechanical effects, or by communication with caverns in the lungs and the bronchi, and in such cases the term bronchial phthisis should be confined.—Understood thus, this form of phthisis is peculiar to children, and attended with very characteristic symptoms; but it is not, as some writers assert, of frequent occurrence.

"The enlarged bronchial glands may act mechanically on the neighboring organs contained in the chest, or they may perforate them. Hence a variety of symptoms, depending on the position or function of the injured part.

"The aorta and pulmonary artery, the vena cava, or the pulmonary veins, may be compressed by the tuberculated glands, and the flow of blood more or less impeded. M. Tonellé has related a case in which the superior cava was completely obstructed, and I have seen one where the pulmonary artery was perfectly flattened between two enormous glands. From the compression of vessels may arise pulmonary appoplexy, fatal hemorrhage, effusions of serum, or symptoms closely resembling those of organic diseases of the heart. The trachea, bronchial tubes, and lungs, may be compressed, and in such cases the symptoms will vary considerably, according to the seat and extent of the mechanical lesion. When the ganglions act on the lower portion of the trachea, M. Rilliet and Barthez have noticed the existence of a loud, sonorous ronchus, which persists for a considerable length of time. In other cases, the pressure on the large bronchial tubes causes more or less feebleness of the respiratory murmur, which is remarkable in being intermittent.

"Pressure on the eighth pair of nerves, or its branches, is often attended by very peculiar modifications of the voice and cough. The former is hoarse or occasionally subdued, and even lost; or the hoarseness and loss of voice may alternate. The cough, also, is frequently hoarse, or occurs in fits which bear a close resemblance to those of hooping-cough, but are not followed by vomiting; or the fits may simulate accesses of asthma, with great oppression of breathing, anxiety, agitation, congestion of the head, and cold, viscid sweats.

"The enlarged or softened glands give rise to another order of symptoms, by perforation of the neighboring parts.—

Thus fatal hemorrhage may arise from perforation of the pulmonary artery; pneumo-thorax from perforation of the lung; difficulty of deglutition and accesses of cough on swallowing from perforation of the œsophagus; but we should observe that these symptoms may equally depend on the presence of tubercular matter or of a cavern in the lungs." 365.

On the subject of diagnosis of this form of phthisis, the author remarks:

"Whenever a child presents several of the rational symptoms of consumption, without our being able to detect any physical signs of the presence of tubercles in the lungs or abdomen, we have good reason to suspect that the bronchial glands are tuberculated. As long as the case continues to present this simple aspect we cannot go beyond suspicion; but it rarely happens that the glands acquire a considerable degree of development, without acting on the surrounding parts or tissues. As these become successively involved, we have a series of varying symptoms, which could not arise from any other source. The eyelids become œdematous, and in proportion to the degree of pressure on the vena cava, the œdema extends to the whole of the face, which is sometimes pale, sometimes tinged with venous injection. This œdema will appear and disappear several times during the course of the disease. The cough suddenly changes its character, and occurs in fits, like those of hooping-cough; the voice gets hoarse, and for days may be altogether lost; fits of asthma or of suffocation, as if the heart were diseased, occur. On examining the chest, we hear a loud sonorous ronchus, which persists for a length of time, and then disappears, or is replaced by other *râles* of an anomalous character. When these symptoms are superadded to the rational signs of phthisis, we can have little hesitation in deciding that they arise from tubercular enlargement of the bronchial glands." 366.

We find nothing new on the subject of treatment. Dr. Green objects to emetics, lest irritation of the abdominal viscera hasten the deposit of tubercle in the abdomen, to which the patients are already too prone.

We much regret that we have been compelled to give so condensed an analysis of this excellent paper. Much, very much as Louis has done, it is evident that he has not exhausted the subject of phthisis, and the accurate information presented to us by Dr. Green will be received as a valuable addition to our history of the disease at the period of infancy. —*Med. Chir. Rev.*

